# A Preliminary Survey of the Distribution of Segmented Filamentous Bacteria in the Porcine Gastrointestinal Tract

**DOI:** 10.1007/s00284-021-02636-0

**Published:** 2021-09-03

**Authors:** Łukasz Grześkowiak, Beatriz Martínez-Vallespín, Jürgen Zentek, Wilfried Vahjen

**Affiliations:** Institute of Animal Nutrition, Freie Universität Berlin, Berlin, Germany

## Abstract

Segmented filamentous bacteria (SFB) are present in various animal species including pigs. The aim of this work was to analyze the occurrence of SFB in different parts of the gastrointestinal tract of piglets of different ages. A total of 377 DNA extracts from stomach, jejunum, ileum, cecum and colon digesta, and from feces collected on different time points, originating from 155 animals, were screened by qPCR method with primers specific for the SFB. SFB sequences were detected in 74 of 377 samples (19.6%) from 155 animals in total. SFB were most abundant in ileum (50.0%), cecum (45.0%), and colon (37.0%), followed by feces (14.6%). SFB prevalence in sows was 12.9% (13/101) and 75.9% (41/54) in individual piglets. Of the 41 SFB-positive piglets, only two samples were from pre-weaning animals, while the rest of samples originated from post-weaning piglets. SFB sequences are abundant in post-weaning piglets, but not in suckling or adult animals. They are most abundant in the ileum and cecum of pigs. Further studies are warranted to reveal the role of SFB in pigs.

## Introduction

The gut microbiota of pigs is characterized by rapid development after birth with certain bacteria temporarily dominating the intestinal tract. Segmented filamentous bacteria (SFB) are Gram-positive, spore-forming bacteria which have been detected in a wide range of mammals, birds, humans, and also in insects [1, 2]. In rodents such as mice and rats, SFB are found predominantly around weaning and persist only shortly afterward [3, 4]. The reason for the age-dependence in SFB colonization is not well understood yet. Although the degree of SFB colonization differs within species, they must be considered as indigenous bacteria of the intestine [5].

Data from light and scanning electron microscopy demonstrate that the main site of SFB colonization in mice, rats, chickens, dogs, and pigs is the ileum, where SFB attach to the intestinal epithelium without provoking an immune response [1, 6, 7]. The unique symbiotic lifestyle of SFB is related to their limited metabolism capacity. For instance, SFB lack the ability to synthesize most amino acids, vitamins, or nucleotides [8] and therefore, they seem to be highly dependent on nutrients and co-factors derived from their host [9]. Since SFB depend on critical growth factors, an in vitro cultivation attempts of SFB remain a challenge [10].

Segmented filamentous bacteria (SFB) are gaining attention due to their role in induction of adaptive and innate immunity in mice and rats [11]. However, recent reports demonstrate that SFB could also be associated with certain autoimmune, chronic inflammatory diseases in mouse models [12]. Thus, their exact role on host health needs still to be clarified.

Although SFB have been widely characterized in the laboratory rodents, still little is known about SFB in pigs. Therefore, the aim of this work was to perform a preliminary survey to detect and analyze the occurrence of SFB in different parts of the gastrointestinal tract of piglets of different age and in the sows’ feces.

## Materials and Methods

### DNA Extracts and qPCR

A total of 377 existing DNA extracts from stomach, jejunum, ileum, cecum and colon digesta, and from feces, originating from 155 animals and 11 different feeding trials, all performed at our animal facility station were collected from the own DNA storage. The samples used in the current study are from the DNA storage and no live animals were used in the current study. All the samples originated from standardized feeding animal trials with identical standard animal husbandry conditions (sows kept in barns; suckling piglets with their mothers until weaning; post-weaning pigs kept in pairs in pens). Animals were fed standard wheat-based diets, appropriate for their age. Since it was a screening study, to avoid the impact of specific dietary treatments on the SFB prevalence, only the DNA from animals assigned to control groups was used (Table 1). The DNA has been obtained using extraction methods with different commercial kits, specifically the QIAamp DNA stool Mini kit (Qiagen, Hilden, Germany) and the NucleoSpin DNA Stool (Macherey–Nagel GmbH and Company KG, Düren, Germany); therefore, the further obtained data were considered as semi-quantitative results. The DNA extracts were subjected to SFB screening by qPCR method with the following primer set: 779F (5′-TGTGGGTTGTGAATAACAAT-3′), 1008R (5′-GCGAGCTTCCCTCATTACAAGG-3′) [13, 14]. The PCR conditions were as follows: 1 × 15 min; 40 × 15 s 95 °C, 60 s 56 °C, 60 s 72 °C; 1 × ramp from 56 to 95 °C. The master mix consisted of Brilliant II SYBR Green QPCR Master Mix with Low ROX (Stratagene, San Diego, CA, USA). Real-time cycler MX3005 and MX3000 (Stratagene) were used for PCR amplification and fluorescent data collection. After confirmation of correct dissociation temperature, samples were identified as positive, if the respective cT-value lay in range of the calibration curve samples (cut-off at 35 cycles).

**Table 1:** Information on the origin of DNA extracts for the study

Item	*N*	Note
Total sample number	377	From a total of 155 animals
Number of trials	11	Only control groups were used
Intestinal location	6	Stomach (*n* = 22), jejunum (*n* = 33), ileum (*n* = 22), cecum (*n* = 20), colon (*n* = 54), feces (*n* = 226)
Piglet samples	276	Suckling piglets (*n* = 107), post-weaned piglets (*n* = 169)
Sow samples	101	All feces
Age groups	9	From 10- to 56-day-old piglets; sows of varying age

### Statistical Analysis

The DNA extracts were not normalized for identical DNA concentration. Instead, inverse cT values from amplification data were used to approximate semi-quantitative distributions of bacteria in sample extracts. As unequal sample sizes were present in the data sets; the non-parametric Kruskal–Wallis test and subsequent Mann–Whitney *U* test were used to denote significant differences. Significance was considered at *P* ≤ 0.05. Data were analyzed in SPSS 24.0.0.0 (IBM, SPSS Statistics, Chicago, IL, USA).

## Results and Discussion

**Fig. 1:**
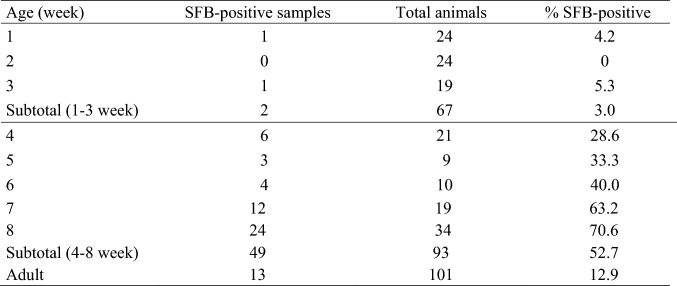
Distribution of animals in which at least one sample (intestinal digesta or feces) was positive for SFB, as analyzed by qPCR. Horizontal line between Subtotal (1–3 week) and 4th week of age indicates weaning and thus separates suckling from weaning piglets

**Fig. 2:**
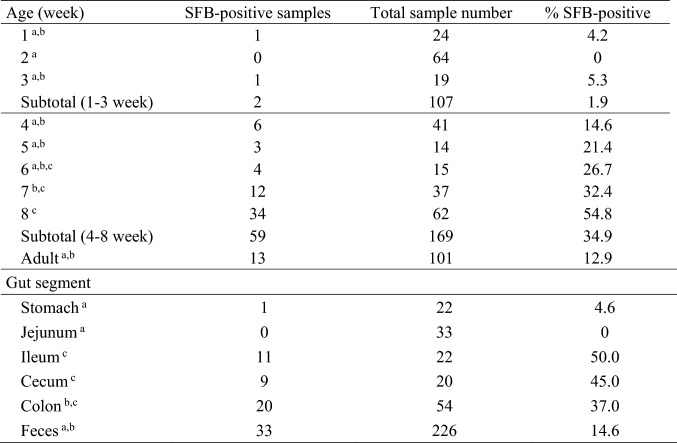
Distribution of SFB-positive samples in the intestinal digesta and feces of pigs according to age and different intestinal segments of pigs, as analyzed by qPCR. Horizontal line between Subtotal (1–3 week) and 4th week of age indicates weaning and thus separates suckling from weaning piglets. ^a,b,c^Different superscripts denote significant differences (% SFB-positive) within a column (*P* ≤ 0.05)

The prevalence of SFB among different animal species seems to be ubiquitous. SFB have been detected in a broad range of mammals like monkeys, horses, cattle, pigs, dogs, mice, rats, and in birds [1–5]. Interestingly, previous attempts to colonize germ-free rats or germ-free mice with SFB obtained from unrelated hosts have failed, indicating that SFB are host specific [15]. Such unique host specificity down to species level may be due to the intimate association of SFB with host epithelial surfaces in the gut. Moreover, SFB have been found to colonize certain species in an age-dependent manner [5, 6]. Here, we show that the prevalence of SFB in pigs is also age dependent. SFB prevalence in the individual piglets was 75.9% (41/54). Specifically, SFB were almost undetectable in suckling piglets (0–5.3%), while their abundance increased after weaning (28.6–63.2%) and diminished drastically in adult pigs (12.9%) (Fig. 1). Further, division of all the analyzed samples (intestinal digesta and feces, different sampling time points) according to piglets or sows yielded a percentage of 22.1% (61/276) SFB-positive samples in piglets and 12.9% (13/101) in sows (Fig. 2). Of the 61 all SFB-positive samples in piglets, only two samples were from a single pre-weaning animal, while the rest came from post-weaning piglets (Fig. 2). Weaning in piglets, which is characterized by transition from sow milk to exclusive solid feeding and separation from mother sows, usually occurs between third and fourth week of life [16]. Our findings are consistent with a previous report on the SFB prevalence in pigs [6]. Also in mice and rats, SFB are almost absent in neonates, while they arise after weaning and diminish again in adulthood [3, 4, 17, 18]. Therefore, it is becoming clear that SFB are more commonly detected in younger versus older animals. However, the rationale for that observation is not understood. In the offspring, the colonization with SFB may depend on numerous factors such as the health status of a mother, and thus timing and persistence of SFB colonization may also be related to the transfer of maternal SFB and certain key features like immune agents. In mammalian neonates including suckling piglets, the specific composition of colostrum and milk, enriched in IgA and other antimicrobial agents, may prevent the SFB from establishing a successful colonization in the gut. Following the weaning period where the cessation of milk ingestion occurs, may open an opportunity for SFB to colonize certain gut niches, as previously investigated in mice and their pups [17]. Even though the SFB abundance increases after weaning, not all weaned piglets are colonized with SFB (Figs. 1, 2). The rationale for differences in colonization between the individual animals is not known yet. Another phenomenon is related to a low abundance of SFB among adult animals including pigs (Fig. 1). When and why the SFB almost vanish when the animal gets older needs further research.

It is known that certain bacteria are able to only temporarily colonize the gut of pigs. For instance, a very short time window of about one week after birth for colonization by *Clostridioides difficile*, including toxin-producing ribotypes, can be observed in healthy neonatal piglets [19, 20]. Most probably, *C. difficile* benefits from a yet immature developing gut microbiota. In healthy suckling piglets, the concentration of *C. difficile* diminishes from the second week onward and the spores or its toxins are rarely detected in weaned or adult pigs [19, 20]. Similarly, various porcine pathogenic *Escherichia coli* strains show different windows of opportunity for colonization and infection in suckling or post-weaning pigs [21, 22]. Whether the phenomenon of colonization resistance is true for SFB is not clear yet, however studies on *C. difficile* and *E. coli* could provide some useful considerations.

Moreover, SFB have been found to induce local immune response in murine gut, specifically activating intestinal IgA or CD4 + Th_17_ cells [23, 24]. The specific strengthening of the host immune system against other commensal bacteria by SFB may help them to compete for niche with other microbes and establish temporal colonization. It seems that the local response of the host does not eliminate the SFB from the gut but only prevents its overgrowth, keeping the SFB immune potential to a minimum necessary need to prevent gut epithelial invasion by pathogens.

Our study provides insight into the topographical distribution of SFB in the intestinal digesta of piglets (Fig. 2). Since we did not perform any dissections of the sows, the distribution of SFB in the digesta of intestinal segments of adult pigs still needs to be characterized. We found that the SFB were most abundant in ileum (50.0%), followed by cecum (45.0%) and colon (37.0%) digesta and feces (14.6%), while they were not detected in the jejunum digesta of piglets (Fig. 2). Previous publications also report the highest abundance of SFB in the ileum of piglets, mice and rats, and in the ileum and cecum of poultry [5, 6, 25]. It seems that the prevalence of SFB in gut segments proximal to the ileum is very low in piglets [6]. Specific physiological conditions in the ileum, such as lower oxygen level, slower transit time, and lower mucus secretion compared to jejunum, may help the SFB attach to the epithelium and establish a temporary population.

Segmented filamentous bacteria (SFB) are spore-forming bacteria that release spores from vegetative cells anchored in the gut epithelium, as previously suggested [1]. The transmission of SFB spores to the offspring is probably via the fecal–oral route. The ingested spores must persist in the gut until conditions are favorable for germination. The localization of SFB spores in the porcine gut before germination has not been confirmed. One could speculate that the SFB spores persist attached in the ileum in which they sense specific factors coming from the jejunum to germinate and release vegetative cells. Based on the total bacterial DNA extracted and primers used, it is not possible to distinguish between vegetative cells and spores of SFB. Finding the proper growth factors would allow to develop suitable media and conditions for in vitro SFB growth.

Segmented filamentous bacteria (SFB) are abundant in post-weaning piglets, but not in suckling or adult animals. SFB are most prominently detected in the ileum and cecum. The presence of SFB depends on unknown parameters during the development of a piglet, e.g., sow milk, solid feeding, immune-related factors, and/or indigenous microbiota. Further studies are warranted to reveal the role of SFB in pigs.
